# Results of caring and reaching for health (CARE): a cluster-randomized controlled trial assessing a worksite wellness intervention for child care staff

**DOI:** 10.1186/s12966-020-00968-x

**Published:** 2020-05-15

**Authors:** Laura A. Linnan, Amber E. Vaughn, Falon T. Smith, Philip Westgate, Derek Hales, Gabriela Arandia, Cody Neshteruk, Erik Willis, Dianne S. Ward

**Affiliations:** 1 Department of Health Behavior, Gillings School of Global Public Health, University of North Carolina at Chapel Hill, CB 7440, Chapel Hill, North Carolina 27599-7440 USA; 2 Center for Health Promotion and Disease Prevention, University of North Carolina at Chapel Hill, Chapel Hill, North Carolina USA; 3 Department of Biostatistics, College of Public Heath, University of Kentucky, Lexington, Kentucky USA; 4 Department of Nutrition, Gillings School of Global Public Health, University of North Carolina at Chapel Hill, Chapel Hill, North Carolina USA

**Keywords:** Worksite, Worker health, Child care, Physical activity

## Abstract

**Background:**

Child care workers are among the lowest paid US workers and experience a wide array of health concerns. The physical and mental demands of their job and the lack of employer-provided health-insurance increase health risks. The Caring and Reaching for Health (CARE) study evaluated a 6-month Healthy Lifestyles intervention targeting child care workers’ physical activity (primary outcome), other health behaviors, and their workplace health environment.

**Methods:**

Eligible child care centers, defined as being in operation for at least 2 years and employing at least four staff, were enrolled into CARE’s cluster-randomized trial. Centers and their child care staff were randomly assigned to either the Healthy Lifestyles (HL) intervention arm or the Healthy Finances (HF) attention control arm using a block randomization approach. Intervention components were delivered through in-person workshops, center-level displays, informational magazines, director coaching, electronic messaging, and an interactive website. Outcome measures were collected during center visits at baseline and immediately post-intervention by trained data collectors blinded to center arm assignment. Workers’ physical activity was assessed with accelerometers, worn for 7 days. Secondary outcome measures included biometric assessments of health and fitness, web-based surveys about health behaviors, and an environmental audit of workplace supports for health. Multi-level linear mixed models assessed worker- and center-level changes in these outcomes.

**Results:**

Participants included 553 child care workers representing 56 centers (HL = 250 staff/28 centers, HF = 303 staff/28 centers). At 6 months, moderate-to-vigorous physical activity declined slightly in both arms (− 1.3 min/day, 95% CI: − 3.0, 0.3 in HL; − 1.9 min/day, 95% CI: − 3.3, − 0.5 in HF), but there was no significant group by time interaction. Several secondary outcomes for other health behaviors and workplace health environment showed improvements in favor of the intervention arm, yet differences did not remain statistically significant after adjustment for multiple comparisons.

**Conclusions:**

While the Healthy Lifestyles intervention did not improve health behaviors or the workplace health environment, results confirmed the pressing need to focus on the health of child care workers. Future interventions should focus on prevalent health issues (e.g., weight, stress), include both high-tech and high-touch intervention strategies, and address work conditions or other social determinants of health (e.g. wages) as a means of improving the health of these essential workers.

**Trial registration:**

Care2BWell: Worksite Wellness for Child Care (NCT02381938).

## Introduction

Low-wage workers are among the fastest growing segments in the United States workforce [1], and they suffer disproportionately from both health behavioral risks factors and chronic diseases that are the leading causes of premature death and disability [2, 3]. While workplace health promotion interventions have achieved some encouraging results on a wide array of behavioral risk factor outcomes such as physical activity, diet, and weight management [4–7]; there is a critical need to develop effective interventions to address the health of low-wage workers [8].

Child care workers are among the lowest paid employees, earning a median hourly wage of $10.72 [9], which is well below the recommended living wage of $16 per hour [10]. Not surprisingly, there is growing evidence that child care workers have heightened prevalence of overweight and obesity, high blood pressure, and diabetes [11–14], placing them at higher risk for many debilitating chronic diseases.

Recently, several advocacy groups have called attention to the health of child care workers. In 2012, Child Care Aware published “Paths to a Healthier Child Care Workforce,” which reported on child care workers’ poor physical activity and dietary habits, identified critical barriers to health and wellness, and suggested health-supportive improvements to the workplace [15]. In 2017, the National Head Start Association released its “Nurturing Staff Wellness Toolkit,” which offered guidance for establishing a staff wellness program and checklist of critical components to include [16].

Despite recognition that child care workers need workplace health promotion efforts, few rigorous evaluations of these efforts exist. Gosliner et al. (2010) conducted a 9-month quasi-experimental study with 13 child care centers in low-income neighborhoods in northern California, and evaluated the impact of adding a worksite wellness program to a training and technical assistance intervention focused on children’s health and nutrition [17]. The worksite wellness program consisted of a kick-off training, monthly newsletters promoting nutrition and physical activity, a walking program, and follow-up visits from intervention staff. Results indicated a modest impact on child care workers’ health behaviors—no significant effect on physical activity and only a small, but significant reduction in sugar sweetened beverage intake. However, the program successfully improved the child nutrition environment and the self-efficacy of staff to communicate with parents about child health. This study was limited by its small sample size (*n* = 82), high staff turnover (23%), and a lack of objective measure of physical activity, health environments and workplace supports.

Another study, completed by Herman et al. (2017) with 75 Head Start sites across five states, evaluated the effectiveness of the “Eat Healthy, Stay Active!” curriculum, a 6-month educational intervention promoting healthier nutrition and increased physical activity among child care workers, parents, and children [18]. Child care workers from these Head Start sites were given materials designed to increase their knowledge of obesity prevention strategies. Information covered dietary guidelines, budget-friendly shopping tips, and ideas for incorporating physical activity into daily routines. Additionally, staff were trained on a core curriculum that they then delivered to parents and children. Curriculum topics included diabetes awareness, obesity prevention, nutrition education, healthy eating on a budget, and physical activity. Results demonstrated a significant reduction in child care workers’ body mass index (BMI) and the percent of workers classified as obese. Significant improvements were also observed in diet and physical activity knowledge and behaviors, but unfortunately, child care worker and parent data for these latter outcomes were reported together. While promising, there was no comparison or control group in this study and outcomes relied only on unvalidated self-report nutrition and physical activity measures.

These studies had important limitations, and both evaluated interventions with a primary target of child health where the child care workers served primarily as key deliverers (not targets) of the intervention. The purpose of the Caring and Reaching for Health (CARE) study was to explore whether a multi-level, theory-guided intervention targeting the child care worker (and the workplace), could improve the health of child care workers. Specifically, this study evaluated the effectiveness of a “Healthy Lifestyles” intervention on child care workers’ physical activity (primary outcome), as well as diet, tobacco and e-cigarette use, sleep, stress, health and biometric indicators (e.g., BMI, blood pressure, cardiovascular fitness, muscle strength), and the workplace health and safety environment (e.g., infrastructure, policies and procedures, programs and promotions, physical environment) compared to an attention control “Healthy Finances” intervention. Study results, as well as implications and recommendations for future worksite-based efforts for child care workers are presented in this paper.

## Methods

The CARE study used a two-arm, cluster-randomized trial to test the effectiveness of a “Healthy Lifestyles” intervention compared to a “Healthy Finances” attention control program. The study was conducted between 2015 and 2018. The study design and protocols for CARE [19] as well as a complete description of the Healthy Lifestyles intervention [20] have been described in accordance with SPIRIT and TIDieR guidelines and published elsewhere. All protocols were approved by the Institutional Review Board at the University of North Carolina at Chapel Hill and registered at www.ClinicalTrials.gov (NCT02381938).

### Participants

A total of 553 child care workers from 56 child care centers located in central North Carolina participated in the study from 2015 to 2019 [19]. A multi-phase recruitment strategy was employed to recruit participants in four waves (i.e. sample size necessitated intervention delivery over four separate time points). Community partners helped introduce child care centers to the study by distributing announcements through existing communication channels and offering group informational sessions. The research team followed-up by phone with centers that expressed interest to review study details and confirm eligibility. In addition, the research team identified child care centers through the North Carolina Division of Child Development and Early Education’s public database of licensed providers [21] and sent announcements about the study directly (by mail and email). Announcements were followed-up with phone calls by the research team to review study details, assess interest, and confirm eligibility. Initial eligibility criteria for centers required they employ at least four staff, have been in operation for at least 2 years, and have no plans to close within the next 18 months. Once initial eligibility was confirmed, research team members conducted onsite center visits to recruit child care workers. To be eligible to participate, workers had to be at least 18 years of age, able to speak and read English, and either pass the Physical Activity Readiness Questionnaire (PAR-Q) screening or obtain medical permission to participate [22]. At least four workers (one administrator and three staff) had to agree to participate and sign consent for the center to remain eligible. In addition, at least three workers (one administration and two staff) had to attend the kick-off event for the center to remain in the study and be randomized.

### Sample size

Based on recent meta-analysis of physical activity interventions [23] and our own pilot data we expected to detect an effect size of 0.30 using a two-sided test of significance. Power analysis indicated randomizing 104 centers and 416 child care staff (4 staff per center) would provide > 80% power to detect the hypothesized between group differences with a type I error rate of 5% with an estimated intra-class correlations (ICC) of 0.02 (based on previous pilot work) [19]. However, monitoring of enrollment showed that both cluster size and rate of attrition were larger than originally assumed. Based on these observations during early waves, a revised power calculation suggested a sample size of 58 centers (26 centers per arm) and 580 child care staff were needed to be enrolled to detect the same effect size.

### Randomization

Workers were randomized in clusters, based on the center where they were employed. Randomization occurred at a kick-off event, which followed baseline data collection. During these events, each center representative selected an envelope from a bowl, and the card within revealed their assignment – Healthy Lifestyles or Healthy Finances. Cards were produced based on randomization tables (generated by the study statistician) created using a block randomization approach. A block size of two was used to ensure balance in the number of centers in each study arm for each of the four waves. Results of randomization were immediately announced. Participating centers (and workers who attended the kickoff event) then adjourned to separate locations to participate in a workshop based on their assignment to Healthy Lifestyles or Health Finances.

### Intervention: healthy lifestyles

The Healthy Lifestyles intervention has been described in detail elsewhere using TIDieR guidelines [19, 20]. Healthy Lifestyles was a six-month, multi-level, theory-guided intervention designed to increase physical activity and improve other health behaviors among child care workers. While Healthy Lifestyles was designed as a workplace-based intervention, its components were used to target three levels of influence from the Social Ecological Framework in order to maximize it potential impact, these included: intrapersonal (individual workers), interpersonal (interactions between co-workers), and organizational (the child care center). Strategies employed to target each level were informed by: Perceptual Control Theory (intrapersonal) [23, 24], Social Support Theory (interpersonal) [25], and Diffusion of Innovation (organizational) [26].

All participants (intervention and control arms) attended a one-day, in-person kick-off event held at a centrally-located community facility (e.g., local church). During the 2-h morning wellness fair, workers visited informational tables manned by local community organizations. The Healthy Lifestyles intervention was launch in the afternoon, when participants attended an 1.5 h educational workshop. In the months that followed, workers participated in three 8-week campaigns (i.e., Every Little Move Counts, Balance Your Menu with Movement, and Moving for a Healthy Life). Each campaign included the following elements: center displays, informational magazines, goal setting and self-monitoring through the CARE website, tailored feedback, prompts, and prize raffles. During each campaign, the center director also received a coaching call from the study interventionist focused on critical elements of workplace health promotion. Table 1 provides a description of each intervention component, including level(s) targeted and theorical underpinnings.

**Table 1:** Healthy Lifestyle intervention components

Intervention Component	SEF Level	Theoretical Guidance	Description
Kick-off event			
Educational workshop	Intrapersonal and Interpersonal	Perceptual Control Theory Social Support Theory	In-person 1.5 h workshop led by the study interventionist and used to raise awareness of current health behaviors vs. national recommendations, distribute pedometers, and introduce reoccurring campaign elements
Campaign elements			
Center displays	Interpersonal	Social Support Theory	Poster and visual materials provided to the director at the outset of each campaign to create/update a bulletin board display where co-workers could read motivational messages, share personal goals, and track group activities
Informational magazines	Intrapersonal	Perceptual Control Theory	Attractive magazines (16–24 pages) distributed to workers that offered information about the benefits of and strategies for improving health behaviors
CARE website – goal setting and self- monitoring	Intrapersonal	Perceptual Control Theory	Interactive website that prompted workers at the beginning of each campaign to set behavior goals—one on physical activity and one on another health behavior; then facilitated weekly logging of self-monitoring information about behavior goals
Tailored feedback	Intrapersonal	Perceptual Control Theory	Automated feedback sent to workers’ email or phone each week by the CARE website summarizing current behaviors and encouraging continued progress toward goals
Prompts	Intrapersonal	Perceptual Control Theory	Automated prompts sent to workers email or phone to remind them about self-monitoring (one per week) or to prompt physical activity (one every other week)
Prize raffles	Intrapersonal	Perceptual Control Theory	Incentives offered to workers that self-monitored and meeting goals (two per campaign)
Director coaching	Organizational	Diffusion of Innovation	One-on-one technical assistance and coaching calls between center directors and the study interventionist (one per campaign) to raise awareness of current workplace supports for health and safety and to set and monitor goals for improving these supports

Two slight modifications were made to the original intervention protocol. First, low compliance with self-monitoring observed in wave 1’s first campaign prompted the addition of a center visit by research staff during the first week of each campaign to remind workers to use their pedometer and log their activity, giving special attention to workers that had not been monitoring. This modification was then implemented in all campaigns across all four waves. Second, the coaching calls were originally designed to be delivered as a group webinar. Scheduling difficulties and lack of attendance in wave 1 made it obvious that individual coaching calls were needed.

### Attention control: healthy finances

The Healthy Finances program was designed to provide a similar level of attention as the Healthy Lifestyles arm, including three, 8-week campaigns with similar components (e.g., center displays, magazine, email/text prompts, prize raffles). The critical difference was that all messages focused on workers’ financial well-being and financial success of the center. Instead of goal-setting and self-monitoring health behaviors, workers were encouraged to take quizzes about the new financial management strategies they learned. Instead of live technical assistance and coaching, center directors were offered pre-recorded webinars on budgeting, marketing strategies for their child care program, and managing legal risk specifically designed for use by child care programs.

### Outcome measures

Outcome measures were collected at three timepoints—baseline, post-intervention (6 months), and maintenance (18 months)—during onsite center visits conducted by trained data collectors. All data collectors were blinded to center arm assignment. Information on group allocation was not provided to data collectors and data collection forms only contained unique identifier codes assigned to each center and participant by the project manager. A full description of data collection protocols and measurement tools is described in detail elsewhere [19]. This paper reports on baseline to immediate post-intervention results only.

#### Primary outcome

Physical activity was assessed using ActiGraph GT3X (ActiGraph, LLC, Pensacola, FL) accelerometers, which workers wore for seven consecutive days. Workers received monitors during center visits along with a postage-paid envelope for their return. Accelerometer data were downloaded using ActiLife software then processed to assess wear and non-wear time. Only participants with valid wear time (i.e., ≥7 h of wear time on ≥4 days) were included in the primary analysis. Adult-specific cut points were then applied to compute minutes of moderate-to-vigorous physical activity (MVPA, ≥2020 counts per minute, primary outcome), lifestyle physical activity (≥760 counts per minute), and sedentary time (≤100 counts per minute) each day [27, 28]. Daily estimates from all valid days of wear were used to calculate average minutes per day for each level of physical activity. To account for variations in wear time, estimates were standardized to a 14-h day (multiplied minutes per hour of sedentary, light, moderate, and vigorous activity by 14). Weekday and weekend day data were also identified and used to calculate average weekday and weekend minutes per day of MVPA.

#### Secondary outcomes

Workers’ health behaviors were self-reported using the Carolina Health Assessment and Research Tool (CHART) [29]. This web-based survey is divided into modules, each of which captures a specific health behavior. CHART was modified for this project to include modules on physical activity, diet, tobacco and e-cigarette use, sleep, and emotional health. CHART also included a demographics module that captured participant demographics and center characteristics. Original CHART items and all modifications drew from existing measures [30–35], as described in detail elsewhere [19]. Drawing on procedures used in source measures, CHART data were summarized to describe health behaviors, specifically times per week of muscle strengthening activities; servings per day of fruit (excluding juice), vegetables, (excluding potatoes), fruits and vegetables (excluding fruit juice and potatoes), sugar sweetened beverages, salty snacks, and fast food; eating habits score (scores range from 0 to 20, higher scores indicates healthier eating habits); current smoking status (smoker or non-smoker) and e-cigarette use (ever used or never used); hours per night of sleep and sleep quality (bad or good); and level of distress (ratings range from 0 to 10, higher scores indicate higher distress).

Biometric assessments of health and fitness indicators were taken by trained data collectors using established protocols. These measures included height, weight, and waist circumference [36]; blood pressure [37]; the six-minute walk test [38]; hand grip [39]; the 30-sec chair sit and stand test [40, 41]; and the four-stage balance test [42, 43]. Height and weight measurements were used to calculate BMI. Blood pressure readings were used to calculate mean arterial pressure.

The workplace health and safety environment was assessed using a tool developed specifically for this study [19], but drawing from existing workplace environmental assessments [44–46]. Information was collected primarily through a structured interview with the director and an environmental observation conducted by data collectors. A scoring rubric was guided by a recent review existing measures of workplace environmental and policy supports for physical activity and healthy eating [47]. Data were then used to calculate scores for four domains: general infrastructure (possible range 0–27), organization policies and procedures (possible range 0–35), programs and promotions (possible range 0–65), and internal physical environment (possible range 0–27). Higher scores always indicated greater support for staff health and safety. For the latter three domains—organization policies and procedures, programs and promotions, and internal physical environment—component scores were also calculated to look at supports available for physical activity (possible range of scores being 0–7, 0–7, and 0–5, respectively) and nutrition (possible range of scores being 0–5, 0–9, and 0–11, respectively).

To document delivery and participation in the intervention, process evaluation measures (dose delivered and received) were collected throughout the study using a combination of direct observation, surveys, and field notes. In addition, 30-min semi-structured interviews were conducted with a small sample of participants (*n* = 30) from the intervention group at the conclusion of the study where we asked them to reflect on their experience in the study. Using purposive sampling, participants from centers (*n* = 10) with the highest and lowest average change in MVPA were recruited for the follow-up interviews. An investigator who was not part of the CARE team with expertise in qualitative research methods conducted all of the interviews, analyzed the data and reported it back to the research team.

### Statistical analysis

Descriptive statistics were used to summarize baseline demographic data for workers and centers in the intervention and control arms. Then, intent to treat (ITT) analyses were performed using all randomized participants. Follow-up assessments were completed with 260 (86%) study participants in the Healthy Finance group and 203 (81%) study participants in the Healthy Lifestyles group. This paper includes 6-month follow-up accelerometer data on the primary outcome from 507 participants at baseline (*n* = 285, HF; *n* = 222, HL) and 379 participants at 6 months (*n* = 221, HF; *n* = 158, HL). The other physical activity measures, and other secondary outcomes we reported on had a different number of missing depending on the outcome variable of interest. Missing data were addressed using maximum likelihood estimation under the assumption of missing at random. Analyses used multi-level linear mixed models (SAS PROC MIXED) for continuous outcomes and GEE-based marginal logistic regression (SAS PROC GLIMMIX) for binary outcomes to examine group differences of primary and secondary outcomes. Models included random cluster effects to account for covariance between participants within the same center as well as fixed effects for time, trial arm, time x arm interaction, and study wave (stratification variable during randomization). An unstructured working covariance was used to account for statistical covariance among repeated measurements from the same subject. The residuals of all continuous outcome variables were checked for normality. Where evidence of departure from normality was apparent the square root of the outcomes were used for analyses in order to obtain valid *p*-values; however, results are presented in their original scale for ease of interpretation.

To assess the impact of missing data, sensitivity analyses were conducted using multiple imputation (SAS PROC MI). Missing data were imputed 50 times for all outcome variables using variables associated with drop-out, demographic variables to be included in later regression analyses, and an indicator for child care center to account for the possibility of clustering. Analyses used linear mixed ANCOVA models to examine change in continuous outcomes and GEE-based marginal logistic regression for binary outcomes. Similar to the ITT analyses, models included random cluster effects to account for covariance between participants within the same center as well as study wave. Models were additionally adjusted for the baseline value of the given outcome variable as well as demographic characteristics identified a priori based on evidence of their predictive value for physical activity and other health behaviors (i.e., age, race, income, baseline BMI). Inferential results based on the imputed data were obtained via SAS PROC MIANALYZE. All tests were two-sided at the 0.05 level. Multiple comparisons for all secondary intervention effects were accounted for by using the false discovery rate method (i.e., the expected proportion of Type I errors among significant findings) to obtain adjusted *p*-values. All analyses were performed using SAS Software, version 9.4 (SAS Institute Inc., Cary, NC).

## Results

### Participant characteristics

**Fig. 1:**
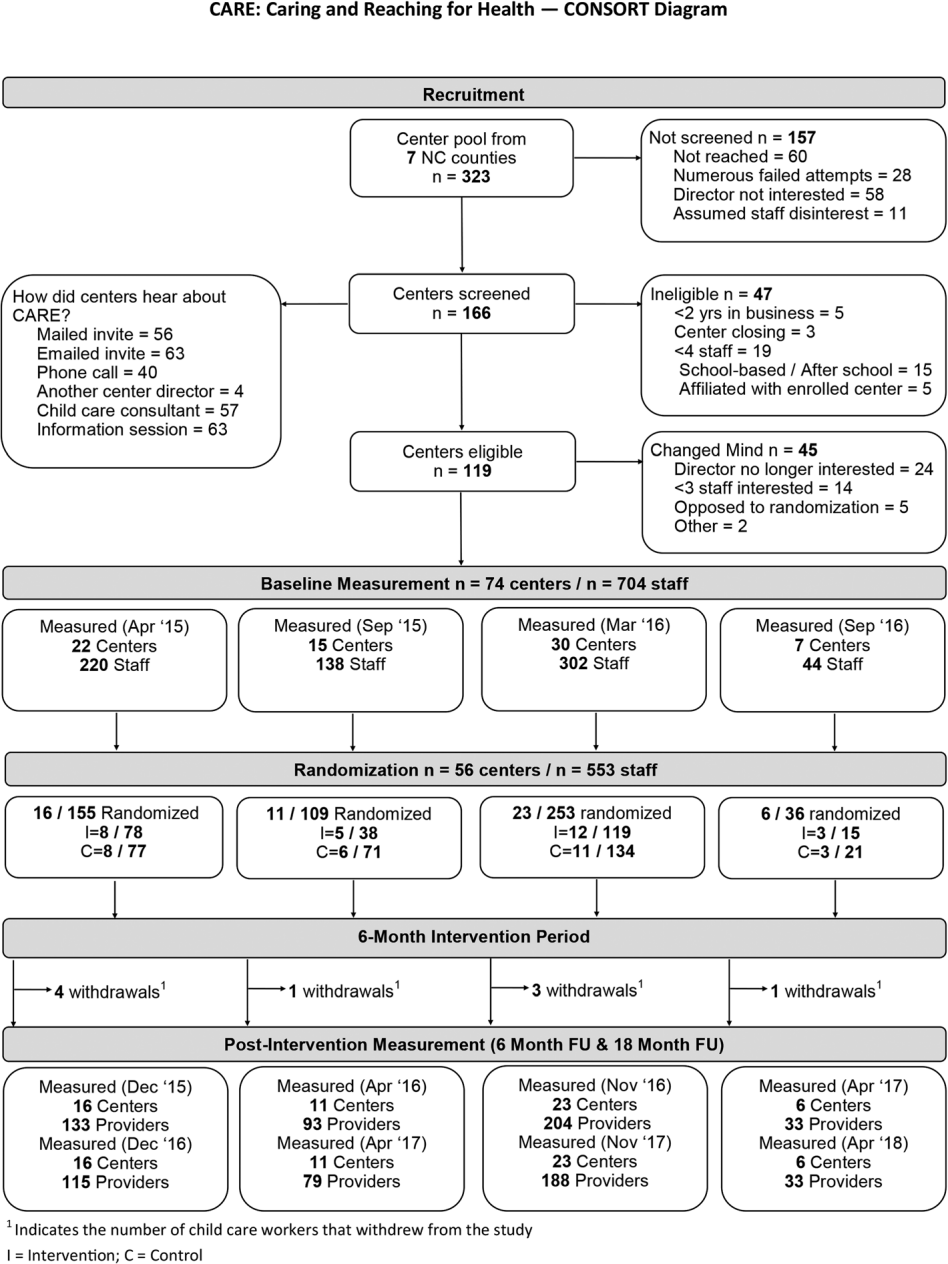
Consort Diagram

The study’s CONSORT diagram is presented in Fig. 1. Across four waves of recruitment, 704 workers representing 74 child care centers were recruited and measured. Of those, 553 workers (78%) were randomized, resulting in 250 workers from 28 centers in the intervention arm and 303 workers from 28 centers in the control arm. The loss of participants between baseline measurement and randomization was anticipated as the study had intentionally included a run-in period that required baseline measurement and center attendance at the kick-off event for a center to be randomized.

Baseline characteristics of participating workers and child care centers are presented in Table 2. Across intervention and control arms, workers were predominantly female (97%) and either African American (51%) or white (37%). Forty percent of workers had a household income less than $20,000 (placing them at or below the federal poverty level for a family of three). Most workers described themselves as staff (82%) versus center administrator. On average, centers employed 15 staff and served 66 children. Centers had, on average, a quality rating of 4.3 stars (based on a rating system of 1–5 stars). In addition, most centers accepted subsidies (96%, financial support for low-income children’s enrollment fees) and were enrolled in the Child and Adult Care Food Program (86%, reimbursement for meals/snacks served to low-income children).

**Table 2:** Baseline characteristics of workers and centers participating in the CARE study

Characteristics	Healthy Lifestyles	Healthy Finances
**Workers**	***n*** **= 250**	***n*** **= 303**
Female (%)	98.8	95.0
Age (years, M ± SD)	40.0 ± 13.1	40.9 ± 13.1
Race and ethnicity (%)		
Non-Hispanic White	39.4	34.6
Non-Hispanic Black	49.8	53.2
Non-Hispanic Other	5.2	5.7
Hispanic	5.6	6.6
Annual household income (%)		
< $20 K	49.1	41.0
> $20 K	50.9	59.0
Highest level of education (%)		
High school diploma/GED	11.6	12.3
Some college	36.8	37.4
Associate degree	25.6	26.5
Bachelor’s degree	22.8	18.5
Graduate, MS, or higher	3.2	5.3
Married/Living with a partner (%)	52.0	52.2
Household size (no. in household, M ± SD)	3.2 ± 1.6	3.3 ± 1.7
Health insured (%)	74.4	79.8
Role at center (%)		
Administrator	21.6	15.6
Staff	78.4	84.5
**Centers**	***n*** **= 28**	***n*** **= 28**
Years in operation (M ± SD)	21.0 ± 14.2	15.5 ± 8.3
Hours per day of operation (M ± SD)	12.7 ± 3.3	13.0 ± 2.7
Enrollment fee ($/week, M ± SD)	142.4 ± 19.9	141.0 ± 17.8
Size		
# of children (M ± SD)	67.1 ± 34.7	65.7 ± 37.6
# of employees (M ± SD)	14.4 ± 8.1	15.0 ± 10.3
Star rating (1–5, M ± SD)	4.3 ± 0.6	4.4 ± 0.8
Privately owned (%)	78.6	64.3
Faith-based (%)	17.9	35.7
Early Head Start (%)	3.6	–
Accepts subsidies (%)	96.4	100
Participates in CACFP (%)	85.7	89.3
NAEYC accredited (%)	21.4	17.9

Post-intervention measures were collected on 463 workers (84%), including 203 workers (81%) in the intervention arm and 260 workers (86%) in the control arm. A comparison of completers versus non-completers revealed that non-completers tended to be younger, lower income, uninsured, and describe themselves as staff. Non-completers also had slightly better health profiles compared to completers (e.g., lower weight, waist circumference, and blood pressure; more steps per day).

### Primary outcome – physical activity

Results of the ITT analyses are presented in Table 3 (for all worker-level outcomes). At baseline, MVPA was significantly lower among participants in the intervention group (16.0 ± 13.4 min/day; *p* = 0.02) compared to those in the control group (18.6 ± 14.8 min/day). Small but significant within group decreases in MVPA were observed in both the intervention group (− 1.3 min/day, *p* = 0.04) and control group (− 1.9 min/day, *p* = 0.001) from baseline to post-intervention. There was no significant between group difference for change in MVPA (*p* = 0.57). Analyses with imputed datasets did not alter results. An ITT analysis of 18-month follow-up data yielded similar results (see Supplemental Tables 1 & 2).

**Table 3:** Results from intent to treat analyses for changes in workers physical activity and other health outcomes from baseline to 6-month follow-up

Outcome	Healthy Lifestyle (*n* = 250)			Healthy Finance (*n* = 303)						
	Baseline mean (SD)	Change mean (95% Cl)	Adj. *p*-value^d^	Baseline mean (SD)	Change mean (95% Cl)	Adj. *p*-value^d^	Diff in mean change	Adj. *p*-value^d^	ICC	ES
**Primary Outcome**										
MVPA (min/day)^a^	16.0 (13.4)	−1.3 (−3.0, 0.3)*	–	18.6 (14.8)	−1.9 (− 3.3, −0.5)**	–	0.6 (− 1.6, 2.8)	–	0.00	0.04
**Secondary Outcomes**										
***Additional Physical Activity Outcomes***										
Lifestyle MVPA (min/day)^a^	117.0 (47.7)	−4.7 (−10.3, 1.0)*	0.05	124.5 (49.7)	−4.2 (−8.9, 0.6)	0.09	−0.5 (−7.9, 6.9)	0.95	0.02	0.01
Sedentary (min/day)	517.4 (74.1)	11.1 (0.6, 21.6)*	0.05	508.8 (74.1)	4.5 (−4.4, 13.3)	0.41	6.6 (−7.1, 20.3)	0.95	0.04	0.09
Weekday MVPA (min/day)^a^	16.9 (14.1)	−1.9 (−3.8, −0.1)**	0.04	20.3 (17.1)	−2.9 (−4.5, − 1.3)***	< 0.001	1.0 (− 1.5, 3.4)	0.95	0.00	0.06
Weekend MVPA (min/day)^a^	12.1 (15.7)	0.3 (−2.5, 3.1)	0.66	13.1 (13.3)	0.9 (−1.5, 3.3)	0.66	−0.6 (−4.3, 3.1)	0.96	0.01	0.04
Meets PA recommendation (150 min/wk.)^b,c^	57 (25.7)	0.64 (0.45, 0.92)*	0.04	93 (32.6)	0.69 (0.52, 0.93)*	0.04	0.92 (0.57, 1.46)	0.95	0.00	0.03
Muscle strengthening activities (# days last week)	1.4 (2.0)	0.7 (0.4, 1.0)***	< 0.001	1.3 (1.9)	0.5 (0.2, 0.8)***	< 0.001	0.2 (−0.2, 0.6)	0.46	0.01	0.10
***Dietary Intake***										
Whole fruit	0.7 (0.6)	0.1 (−0.02, 0.2)	0.13	0.8 (0.7)	−0.1 (− 0.2, 0.01)	0.15	0.2 (0.02, 0.3)*	0.10	0.01	0.30
Vegetable (excluding potatoes)^a^	1.0 (0.7)	0.1 (0.03, 0.3)*	0.04	1.0 (0.8)	−0.02 (−0.1, 0.1)*	0.05	0.2 (0.01, 0.3)*	0.10	0.02	0.26
Fruits &vegetables (excluding potatoes and juice)^a^	1.6 (1.1)	0.2 (0.04, 0.4)*	0.04	1.8 (1.2)	−0.1 (−0.3, 0.1)	0.26	0.3 (0.1, 0.6)**	0.06	0.02	0.26
Sugar sweetened beverage^a^	1.8 (1.9)	−0.5 (−0.8, − 0.2)***	< 0.001	1.6 (1.8)	− 0.2 (− 0.5, 0.03)	0.15	−0.3 (− 0.7, 0.1)*	0.11	0.01	0.16
Salty snack^a^	1.3 (1.5)	−0.5 (− 0.7, − 0.2)***	< 0.001	1.4 (1.8)	−0.3 (− 0.5, − 0.0)**	0.02	−0.2 (− 0.5, 0.2)	0.32	0.00	0.12
Fast food/ Eating out^a^	0.4 (0.4)	−0.1 (− 0.2, − 0.1)***	< 0.001	0.3 (0.3)	−0.1 (− 0.1, − 0.1)*	0.04	−0.1 (− 0.1, − 0.02)**	0.06	0.00	0.28
Eating Habits Score	9.1 (3.3)	1.2 (0.8, 1.8)***	< 0.001	9.6 (3.4)	0.4 (−0.1, 0.8)	0.15	0.9 (0.2, 1.6)**	0.06	0.00	0.27
***Tobacco and e-cigarette Use***										
Smoking status (current smoke/non-smoker)^b,c^	38 (15.2)	0.66 (0.46, 0.92)*	0.03	40 (13.3)	1.05(0.81, 1.37)	0.74	0.6 (0.4, 1.0)*	0.10	0.04	0.04
E-cig use (ever used/never used)^b,c^	25 (10.0)	0.73 (0.45, 1.20)	0.24	28 (9.3)	0.68 (0.40, 1.19)	0.25	1.1 (0.5, 2.2)	0.87	0.04	0.11
***Sleep***										
Hours/night	6.3 (1.4)	0.4 (0.2, 0.5)**	< 0.001	6.4 (1.4)	0.1 (−0.1, 0.3)	0.31	0.3 (0.01, 0.5)*	0.11	0.03	0.21
Quality (good/bad)^b,c^	190 (76.0)	1.38 (0.99, 1.92)	0.07	236 (78.4)	0.98 (0.76, 1.26)	0.87	1.4 (0.9, 2.1)	0.20	0.03	0.02
***Stress***										
Perceived level of distress	3.9 (2.7)	−1.2 (−1.6, −0.7)***	< 0.001	4.0 (2.8)	−0.7 (−1.1, − 0.3)*	< 0.01	−0.5 (−1.1, 0.1)	0.20	0.00	0.18
***Health and Fitness Indicators***										
BMI (kg/m^2^)	33.7 (9.1)	0.1 (−0.2, 0.4)	0.39	34.2 (8.8)	0.0 (−0.2, 0.3)	0.82	0.1 (−0.3, 0.5)	0.78	0.02	0.01
Waist circumference (cm)	104.6 (18.6)	0.6 (−0.3, 1.6)	0.22	106.1 (17.9)	1.1 (0.3, 1.9)*	0.03	−0.5 (−1.7, 0.8)	0.64	0.01	0.03
Mean arterial pressure	94.2 (14.0)	−1.8 (−3.5, −0.1)*	0.05	95.0 (13.2)	−3.6 (−5.1, −2.2)***	< 0.001	0.8 (− 0.4, 4.0)	0.20	0.07	0.06
6 min walk (m)	429.0 (78.3)	−13.5 (− 30.0, − 1.0)*	0.05	456.1 (79.8)	−12.1 (− 22.3, − 1.9)*	0.04	−1.3 (− 14.5, 14.8)	0.87	0.11	0.02
Grip strength (kg)	26.3 (9.3)	1.9 (0.4, 3.5)*	0.03	28.5 (9.1)	1.6 (0.3, 2.9)	0.04	0.3 (−1.7, 2.4)	0.86	0.07	0.03
Chair sit and stand (reps)	14.9 (4.3)	−1.5 (−2.2, −0.8)***	< 0.001	15.2 (4.6)	− 1.3 (− 1.8, − 0.7)***	< 0.001	−0.2 (− 1.1, 0.7)	0.78	0.08	0.04
Highest phase completed in balance test^b,c^	99 (76.7)	1.01 (0.68. 1.52)	0.93	151 (77.4)	1.11 (0.67, 1.86)	0.74	0.9 (0.5, 1.7)	0.86	0.01	0.01
***Teacher Physical Activity Practices***										
Total min PA provided^a^	170.8 (122.3)	−10.5 (−33.9, 13.0)	0.87	162.7 (117.0)	5.3 (−14.9, 25.5)	0.70	−15.7 (−46.7, 15.2)	0.71	0.07	0.13
Total min teacher-led PA^a^	70.4 (55.9)	−2.5 (−16.9, 12.0)	0.87	67.4 (69.2)	−0.1 (−12.3, 12.3)	0.73	−2.4 (− 21.4, 16.6)	0.71	0.00	0.04
Outside time as reward score	3.4 (0.7)	−0.0 (− 0.2, 0.1)	0.87	3.4 (0.7)	− 0.0 (− 0.1, 0.1)	0.88	−0.0 (− 0.2, 0.2)	0.99	0.00	0.00
TV as reward score^b,c^	165 (72.1)	1.31(0.89, 1.90)	0.48	119 (67.2)	0.74 (0.48, 1.12)	0.33	1.77 (1.01, 3.12)	0.41	0.13	0.03
Praise PA score^b,c^	82 (46.3)	1.02 (0.78, 1.34)	0.87	90 (39.5)	1.21 (0.88, 1.66)	0.36	0.85 (0.56, 1.29)	0.71	0.00	0.02
Modeling PA score	3.7 (0.7)	0.2 (0.0, 0.3)	0.06	3.7 (0.8)	0.2 (0.1, 0.3)**	< 0.01	−0.0 (−0.2, 0.1)	0.93	0.06	0.00
Encourage/Prompt PA score	3.9 (0.7)	−0.0 (− 0.1, 0.1)	0.87	3.9 (0.7)	0.1 (− 0.0, 0.2)	0.36	− 0.1 (− 0.2, 0.1)	0.71	0.04	0.14
PA education score	3.5 (0.9)	0.2 (0.0, 0.3)	0.06	3.4 (0.9)	0.2 (0.1, 0.3)**	0.01	−0.0 (− 0.2, 0.2)	0.99	0.04	0.00
Supportive PA environment score	3.6 (0.8)	0.1 (−0.1, 0.2)	0.68	3.6 (0.8)	0.2 (0.0, 0.3)*	0.04	−0.1 (− 0.3, 0.1)	0.71	0.05	0.12

*PA* physical activity, *MVPA* moderate-vigorous PA

^a^Square root transformation of the outcomes were used for analyses and thus providing valid *p*-values, however, results are presented in their original scale

^b^Results for baseline are presented as n (%)

^c^Results for change are presented as OR (95% CI)

^d^Adjusted *p*-values accounting for multiple comparisons using the false discovery rate method

Unadjusted significance *p < 0.05, **p < 0.01, ***p < 0.001

There were no significant within or between group differences for change in sedentary activity, lifestyle physical activity, or weekend MVPA. Small but significant within group decreases were seen in weekday MVPA in both the intervention group (− 1.9 min/day, *p* = 0.04) and control group (− 2.9 min/day, *p* < 0.001) from baseline to post-intervention. Odds of meeting physical activity recommendations also significantly decreased within both the intervention group (− 37, 95% CI, 8–66%) and control group (− 31, 95% CI, 7–48%) from baseline to post-intervention. In contrast, small but significant within group increases were seen in days per week of muscle strengthening activities in both the intervention group (0.7 times/week, *p* < 0.001) and control group (0.5 times/week, *p* < 0.001). There were no significant between group differences in these additional physical activity outcomes. Analyses with imputed data did not change these results.

### Secondary outcomes

#### Health behaviors

Prior to adjustment for multiple comparisons, significant modest improvements in several health-related behaviors were observed from baseline to post-intervention in the intervention group compared to the control group. Improvements included intake of fruits and vegetables (+ 0.3 times/day, *p* < 0.01), sugar sweetened beverages (− 0.3 times/day, *p* = 0.04), eating out (− 0.1 times/day, *p* < 0.01), and overall eating habits score (+ 0.9 points, *p* < 0.01). Similarly, hours of sleep/night (+ 0.3 h/day, *p* < 0.046) and smoking status (OR = 0.60, *p* = 0.03) showed modest, but statistically significantly improvements from baseline to post-intervention in the intervention vs. control group. After adjusting for multiple comparisons, these between group differences were no longer significant. Analyses with imputed data showed significant improvement in only dietary intake of salty snacks (− 0.3 times/day, *p* = 0.016) in the intervention group compared to the control group. Once again, differences were no longer significant after adjusting for multiple comparisons.

#### Workplace health and safety

Workplace health and safety results using ITT analyses are presented in Table 4. Prior to adjustment for multiple comparisons, there were significant increases in the physical activity component scores in both the organization policies and procedures domain (*p* = 0.048) and the programs & promotions domain (*p* = 0.01) in favor of intervention centers. However, these between group differences were no longer significant after adjusting for multiple comparisons. In analyses with imputed data, only the change in physical activity component score for the programs and promotions domain was significant (*p* = 0.02), until adjusted for multiple comparisons.

**Table 4:** Results from intent to treat analyses for changes in child care centers’ workplace health and safety environmental supports from baseline to 6-month follow-up

Outcome	Healthy Lifestyle (*n* = 28)			Healthy Finance (n = 28)					
	Baseline mean (SD)	Change mean (95% Cl)	Adj. *p*-value^1^	Baseline mean (SD)	Change mean (95% Cl)	Adj. *p*-value^1^	Diff in mean change	Adj. *p*-value^1^	ES
***Worksite Health and Safety***	41.8 (11.9)	0.9 (−4.1, 5.9)	0.71	44.0 (13.1)	1.0 (−2.5, 4.4)	0.91	−0.0 (−6.0, 5.9)	0.99	0.00
Infrastructure score	9.7 (4.2)	−0.8 (− 2.7,1.1)	0.54	9.8 (3.7)	0.1 (−1.7,1.9)	0.91	−0.9 (−3.5,1.7)	0.85	0.22
Organization Policies & Procedures (OPP)	11.5 (3.6)	1.5 (0.4, 2.6)*	0.04	12.3 (4.1)	0.7 (−0.5, 2.0)	0.91	0.8 (−0.9, 2.4)	0.85	0.21
OPP - Physical activity score	1.6 (1.0)	1.0 (0.5, 1.5)***	< 0.01	2.1 (1.3)	0.4 (−0.1, 0.8)	0.91	0.6 (0.01, 1.3)*	0.26	0.52
Programs & Promotions (PP)	7.4 (5.0)	0.6 (−1.9, 3.0)	0.70	8.4 (5.8)	0.2 (−1.3, 1.7)	0.91	0.4 (−2.5, 3.2)	0.98	0.07
PP - Physical activity score	0.5 (0.8)	0.8 (0.3, 1.3)**	0.01	0.5 (1.0)	0.1 (−0.3, 0.4)	0.91	0.7 (0.1, 1.3)*	0.11	0.77
Internal Physical Environment (IPE)	13.2 (1.8)	−0.3 (−1.2, 0.6)	0.65	13.5 (2.2)	−0.1 (− 0.8, 0.7)	0.91	− 0.2 (− 1.4, 1.0)	0.98	0.10
IPE - Physical activity score	0.7 (0.9)	0.4 (−0.0, 0.8)	0.14	0.9 (1.1)	0.1 (−0.2, 0.4)	0.91	0.3 (−0.2, 0.8)	0.85	0.30

*SD* standard deviation, *CI* confidence interval, *Adj.* adjusted

Unadjusted significance * *p* < 0.05, ** *p* < 0.01, *** *p* < 0.001

^1^Adjusted *p*-values accounting for multiple comparisons using the false discovery rate method

### Process evaluation

Process evaluation data are presented in Table 5, confirming that the intervention was delivered as intended. However, workers’ engagement with various intervention components was low.

**Table 5:** Intervention Delivery and Participation

Intervention Component	Delivery by Research Team	Participation by Centers (*n* = 28) & Workers (*n* = 250)
Educational workshops	7 sessions offered as part of kick-off events; most waves were offered a choice of 2 dates.	100% of centers were represented at workshops; only 54% of workers attended
Magazines	Magazines were delivered during the first week of each campaign to all 28 centers.	While magazines were sent to each center, we did not collect data asking participants to recall if they received the magazine from the center director.
Self-monitoring	Pedometers and website access were made available to 100% of workers either during the educational workshop or a follow-up center visit (for those who did not attend the workshop).	72% of workers monitored at least once
Prompts	Test accounts created by research staff monitored delivery of messages 1 week ahead of time. All problems were either resolved prior to scheduled distribution or prompts were sent manually.	While we monitored test accounts to ensure messages were delivered to our staff as scheduled, we do not have verification that all workers received all messages
Feedback	Test accounts also used to monitor delivery of tailored feedback.	While we monitored test accounts to ensure delivery of tailored feedback to our staff as scheduled, we do not have verification that all workers received all tailored feedback
Raffle	Raffles were completed for each wave and each campaign.	Only 44% of workers qualified for entry into at least one raffle
Center Visuals	All 28 centers were provided with center visuals during the initial workshop; and all 28 centers received updated materials at the beginning of campaigns 2 and 3.	67% of centers had visuals displayed when visited
Director Coaching	During wave 1 we hosted 4 webinars and invited all 8 directors in that wave to attend. During waves 2–4, all 20 directors were offered director coaching calls.	During wave 1, 100% of directors participated in at least 1 webinar. During wave 2–4, 100% of directors participated in at least 1 coaching call

## Discussion

The CARE study is the first randomized controlled trial of a workplace health promotion intervention focusing on the health of the child care workers and their workplace environment. The Healthy Lifestyles intervention targeted multiple levels of influence and integrated theory to inform strategies used at each level, yet it failed to improve physical activity, the primary target. In fact, workers in both groups has slightly decreased levels of physical activity.

Workers in the Healthy Lifestyles intervention had significant improvements in several dietary variables (i.e., fruit and vegetables, sugar sweetened beverages, eating out, and overall eating habits score) and a decreased odd (34%) of smoking. These results were similar to those observed in our pilot [20]. While encouraging, results must be interpreted with caution as they were no longer statistically significant after adjusting for multiple comparisons and in sensitivity analyses using imputed data.

The multi-level, theory-guided Healthy Lifestyles intervention integrated proven behavior change strategies [23] and effective intervention elements [48]. A meta-analysis of healthy eating and physical activity interventions suggested that self-monitoring along with at least one other behavioral control technique (e.g., goal setting, performance feedback) significantly improved outcomes [23]. Based on this research, a centerpiece of the Healthy Lifestyles intervention was weekly goal setting, self-monitoring, and tailored feedback facilitated through the CARE website. Further, a systematic review of 20 workplace physical activity studies noted that successful interventions tended to be shorter in duration (< 6 months), and use pedometers, internet-based approaches, and social and environmental changes [48]. Hence, the CARE intervention incorporated many similar elements. However, a 2014 systematic review of workplace physical activity interventions revealed that only 32 of 58 studies produced statistically significant improvements [49]. Thus, specific intervention strategies for improving physical activity in the context of workplace interventions, particularly for low wage workers, require additional research.

Clearly, the Healthy Lifestyles intervention was not effective in producing significant improvements in the primary outcome (physical activity) when the HL group was compared with the HF group. These results warrant consideration of several other plausible explanations: a potential lack of engagement among workers with critical intervention components, the lack of intervention intensity, quality or duration, and/or the inability of workers to overcome competing demands. With regard to the lack of engagement, process evaluation data demonstrated that child care workers did not consistently engage in self- monitoring, a critical component of the intervention. While 72% of workers in the Healthy Lifestyles arm logged their steps at least once, far fewer logged the steps regularly as only 44% qualified for raffle entry (which required regular monitoring). Thus, lack of engagement with the intervention may have led to non-significant results.

It is also possible that the intervention had insufficient intensity, quality or duration. Though we had positive pilot study results, it is possible that promoting a relatively low intensity “move more” approach to physical activity such as walking did not appeal to these workers. The six-month intervention duration was effective in previous studies [48], but post-intervention interviews with child care workers revealed a strong desire for more personal interaction that was “intensive” and “high-touch”. For example, while the messages and feedback were delivered to individuals through text or email, participants desired more personal communications or additional events with coworkers and/or research staff, similar to the initial kickoff events. The center visuals were intended to leverage social support from coworkers by promoting team building and group physical activity. However, center visuals were displayed in only 67% of centers, and often were taken down following the first campaign. Child care workers are likely to need more assistance from research staff or center leadership to utilize these center visuals to their full potential.

Another potential explanation for the lack of change in physical activity is that child care workers face serious competing demands which limit physical activity that our Healthy Lifestyles intervention could not overcome. As emphasized in a white paper produced by Child Care Aware, child care workers report being very tired at the end of the day, having limited time, and facing a number of competing after-work responsibilities (e.g., caring for their own children, preparing meals, maintaining the household or holding secondary jobs), as well as being unable to afford memberships in fitness centers and gyms [15]. Baseline levels of stress among child care workers in this study were significantly higher than stress levels among the general population [11, 19]. The aforementioned challenges child care workers face are exacerbated by having low wages and physically demanding jobs. Thus, any single or combination of these reasons may explain why the Healthy Lifestyles intervention did not produce intended improvements in physical activity among child care workers in this study.

Despite a lack of effect on physical activity for the individual worker, results showed some promising changes in workplace supports for physical activity, particularly in policies, procedures, programs, and promotions. These environmental changes align well with the content of the coaching sessions which encouraged center directors to expand opportunities to support physical activity through group activities, education, and policy changes. For example, it may be helpful to consider purchasing equipment such as treadmills or recumbent bicycles or weight training equipment and encouraging workers to use it before/after work or during breaks as one way to increase access in the work environment. The content of the coaching calls were also consistent with several of the essential elements for staff wellness recommended by the National Head Start Association, such as identifying a wellness leader, assigning a wellness committee, and offering engaging wellness activities [16]. However, the three coaching calls offered as part of Healthy Lifestyles may not have been enough to focus on other recommended elements, such as conducting a detailed needs assessment, identifying useful resources and partnerships, communicating effectively, and having a continuous evaluation plan [45].

Results of this study and similar recent workplace trials [6, 7] suggest that improving workers’ health will require a more comprehensive approach that addresses not only health behaviors but also the work environment, working conditions, benefits and compensation [48–50]. Child care workers are confronted with many challenges on a daily basis from both their job and life that may leave them little discretionary time or energy to focus on their health. Providing health benefits and/or improving compensation and wages of these important members of the workforce may provide a more direct and lasting pathway to improved health than health promotion alone. Otten et al. (2019) are studying the effects of a policy to improve wages among child care workers in Seattle [51]. Their baseline findings reiterate the poor benefits and lack of compensation as well as the emotional strains and societal disrespect experienced by these workers [51]. In this study with low wage child care workers, it is interesting that the Healthy Finances intervention which focused on budgeting, securing credit, and managing personal finances appeared equally effective in reducing stress as did the Healthy Lifestyles intervention which included content on traditional stress management techniques. Given the growing body of literature documenting the high prevalence of stress and depression among child care workers [11, 14, 51, 52], future research is warranted on the underlying causes of child care worker stress (including from the work environment), the ways in which stress can be reduced, and the impact of stress reduction on overall health. A Total Worker Health approach [53–55] that addresses both working conditions (e.g. wages, work schedules, etc.) and health behaviors may be more impactful than traditional health promotion activities, particularly for low-wage workers like those in child care [56, 57].

### Strengths and limitations

This study had many strengths, including the use of a multi-level, theory-guided intervention that had been previously proven effective in pilot-testing, an attention control (Healthy Finances) group, an objective assessment of physical activity (accelerometry) with excellent follow-up, interview data with participants post-intervention, and, a rigorous analytic approach including intent-to-treat and multiple imputation techniques for missing data. The study was led by an experienced team with long-standing connections with the child-care community, who were enthusiastic participants in the research. Despite these strengths, we know that the use of conservative analytic approaches (e.g., intent-to-treat analysis, controlling for potential confounders) can minimize effect sizes [58] and while we were powered to detect a 0.03 effect, we enrolled slightly below the desired number of child care staff and centers, which may have reduced our power. Since centers were recruited from seven counties in NC, results cannot be generalized to all child care centers in the state or nation. Further, the intervention was delivered to child care workers who volunteered to participate, and thus, may not be representative of all child care workers. And, while we collected a wide array of process evaluation data to help with interpretation of our data, we did not get sufficient tracking data on several indicators of program receipt among participants. Despite these issues, we believe that the many study strengths outweigh its limitations, particularly in light of the fact that this was among the very first trials addressing the health of child care workers.

## Conclusions

We conducted one of the first and most rigorous evaluations of a multi-level, theory-guided intervention designed to increase physical activity among child care workers. There were some encouraging, albeit modest, positive changes documented at the child care worker level on muscle strengthening activities and several secondary health behavior outcomes (e.g. eating behaviors, sleep and tobacco use), but not on the primary physical activity outcome. There were also some positive changes at the center level in terms workplace supports for physical activity. However, results often did not remain significant after adjusting for multiple comparisons or using multiple imputation to address missing data. Lessons were learned about the challenges facing child care workers, especially the competing demands and work conditions that make it difficult for child care workers to fully engage in a workplace physical activity intervention. To help promote physical activity and improve the health and well-being of child care workers, future research should consider better understanding the causes of the high levels of stress they report, developing more personalized, high-touch interventions; and considering a Total Worker Health approach which addresses the health of the worker, demanding working conditions and the larger social context in which the child care worker exists.

## Supplementary information

**Additional file 1: Table S1.** Results from intent to treat analyses for changes in workers physical activity and other health outcomes from baseline to 18-month follow-up. **Table S2.** Results from intent to treat analyses for changes in child care centers’ workplace health and safety environmental supports from baseline to 18-month follow-up.

## Data Availability

Datasets used and/or analyzed during the current study are available from the corresponding author on reasonable request provided the investigator who proposes to use the data has approval from an Institutional Review Board (IRB), Independent Ethics Committee (IEC), or Research Ethics Board (REB), as applicable, and executes a data use/sharing agreement with UNC.

## Abbreviations

ANCOVA: Analysis of covariance
BMI: Body mass index
CARE: Caring And Reaching for Health
CHART: Carolina Health Assessment and Research Tool
GEE: Generalized estimating equation
ITT: Intent to treat
MVPA: Moderate-to-vigorous physical activity
PARQ: Physical Activity Readiness Questionnaire
