# Supplementary material for: Results of caring and reaching for health (CARE): a cluster-randomized controlled trial assessing a worksite wellness intervention for child care staff
Source: Int J Behav Nutr Phys Act. 2020 May 15;17:64. doi: 10.1186/s12966-020-00968-x (PMC7227251; doi:10.1186/s12966-020-00968-x)
Supplement: Supplementary file 1 — Additional file 1: Table S1. Results from intent to treat analyses for changes in workers physical activity and other health outcomes from baseline to 18-month follow-up. Table S2. Results from intent to treat analyses for changes in child care centers’ workplace health and safety environmental supports from baseline to 18-month follow-up. [file 12966_2020_968_MOESM1_ESM.docx]

| **Supplementary Table 1.** Results from intent to treat analyses for changes in workers physical activity and other health outcomes from baseline to 18-month follow-up. | | | | | | | | | | | |  |
| --- | --- | --- | --- | --- | --- | --- | --- | --- | --- | --- | --- | --- |
|  | **Healthy Lifestyle**  **(n=250)** | | |  | **Healthy Finance**  **(n=303)** | | |  |  |  |  |  |
|  | **Baseline** | **Change** | **Adj.** |  | **Baseline** | **Change** | **Adj.** | **Diff in mean change** | **Adj.** |  |  |  |
| **Outcome** | **mean (SD)** | **mean (95% Cl)** | **p-value^4^** |  | **mean (SD)** | **mean (95% Cl)** | **p-value^4^** |  | **p-value^4^** | **ICC** | **ES** |  |
| **Primary Outcome** |  |  |  |  |  |  |  |  |  |  |  |  |
| MVPA (min/day)^1^ | 16.0 (13.4) | 0.3 (-1.8, 2.3) | - |  | 18.6 (14.8) | -2.3 (-4.1, -0.5)** | - | 2.6 (-0.1, 5.2) | - | 0.00 | 0.18 |  |
| **Secondary Outcomes** |  |  |  |  |  |  |  |  |  |  |  |  |
| ***Additional Physical Activity Outcomes*** |  |  |  |  |  |  |  |  |  |  |  |  |
| Lifestyle MVPA (min/day)^1^ | 117.0 (47.7) | -0.9 (-7.8, 6.1) | 0.717 |  | 124.5 (49.7) | -6.3 (-12.4, -0.3)* | 0.114 | 5.5 (-3.8, 14.7) | 0.453 | 0.02 | 0.11 |  |
| Sedentary (min/day) | 517.4 (74.1) | 14.0 (2.5, 25.6)* | 0.077 |  | 508.8 (74.1) | 9.9 (-0.1, 19.9) | 0.136 | 4.2 (-11.1, 19.4) | 0.717 | 0.04 | 0.06 |  |
| Weekday MVPA (min/day)^1^ | 16.9 (14.1) | -0.1 (-2.4, 2.3) | 0.717 |  | 20.3 (17.1) | -3.0 (-5.0, -1.0)** | 0.018 | 2.9 (-0.2, 6.0) | 0.170 | 0.00 | 0.19 |  |
| Weekend MVPA (min/day)^1^ | 12.1 (15.7) | 1.0 (-1.8, 3.8) | 0.231 |  | 13.1 (13.3) | -0.9 (-3.4, 1.5) | 0.453 | 1.9 (-1.8, 5.6) | 0.170 | 0.01 | 0.13 |  |
| Meets PA recommendation (150min/wk.)^2,3^ | 57 (25.7) | 0.99 (0.70, 1.39) | 0.936 |  | 93 (32.6) | 0.67 (0.48, 0.96)* | 0.101 | 1.46 (0.90, 2.39) | 0.225 | 0.00 | 0.21 |  |
| Muscle strengthening activities (# days last week) | 1.4 (2.0) | 0.4 (0.1, 0.7)** | 0.042 |  | 1.3 (1.9) | 0.5 (0.2, 0.8)*** | 0.007 | -0.1 (-0.5, 0.3) | 0.778 | 0.01 | 0.04 |  |
| ***Dietary Intake*** |  |  |  |  |  |  |  |  |  |  |  |  |
| Whole fruit | 0.7 (0.6) | 0.1 (-0.0, 0.2) | 0.292 |  | 0.8 (0.7) | 0.0 (-0.1, 0.1) | 0.898 | 0.1 (-0.1, 0.2) | 0.734 | 0.01 | 0.09 |  |
| Vegetable (excluding potatoes)^1^ | 1.0 (0.7) | -0.04 (-0.2, 0.1) | 0.735 |  | 1.0 (0.8) | 0.0 (-0.2, 0.1) | 0.282 | 0.0 (-0.1, 0.2) | 0.898 | 0.02 | 0.04 |  |
| Fruits &vegetables (excluding potatoes and juice)^1^ | 1.6 (1.1) | 0.7 (-0.1, 0.2) | 0.201 |  | 1.8 (1.2) | 0.5 (-0.2, 0.1)* | 0.158 | 0.5 (-0.2, 0.3) | 0.282 | 0.02 | 0.41 |  |
| Sugar sweetened beverage^1^ | 1.8 (1.9) | -0.3 (-0.6, -0.0)** | 0.084 |  | 1.6 (1.8) | 0.0 (-0.3, 0.3) | 0.992 | -0.3 (-0.7, 0.1)* | 0.158 | 0.01 | 0.19 |  |
| Salty snack^1^ | 1.3 (1.5) | -0.1 (-0.4, 0.2) | 0.880 |  | 1.4 (1.8) | -0.1 (-0.4, 0.1) | 0.734 | 0.0 (-0.3, 0.4) | 0.939 | 0.00 | 0.03 |  |
| Fast food/ Eating out^1^ | 0.4 (0.4) | -0.1 (-0.1, -0.0)** | 0.084 |  | 0.3 (0.3) | 0.0 (-0.0, 0.1) | 0.311 | -0.1 (-0.2, -0.0)** | 0.084 | 0.00 | 0.26 |  |
| ***Tobacco and e-cigarette Use*** |  |  |  |  |  |  |  |  |  |  |  |  |
| Smoking status (current smoke/non-smoker)^2,3^ | 38 (15.2) | 0.9 (0.5, 1.5) | 0.880 |  | 40 (13.3) | 1.0 (0.6, 1.4) | 0.898 | 0.9 (0.5, 1.8) | 0.898 | 0.04 | 0.00 |  |
| E-cig use (ever used/never used)^2,3^ | 25 (10.0) | 1.0 (0.7, 1.3) | 0.898 |  | 28 (9.3) | 1.0 (0.6, 1.7) | 0.939 | 0.9 (0.5, 1.7) | 0.898 | 0.04 | 0.00 |  |
| ***Sleep*** |  |  |  |  |  |  |  |  |  |  |  |  |
| Hours/night | 6.3 (1.4) | 0.3 (0.1, 0.4)* | 0.118 |  | 6.4 (1.4) | 0.0 (-0.2, 0.2) | 0.898 | 0.2 (-0.0, 0.5) | 0.274 | 0.03 | 0.16 |  |
| Quality (good/bad)^2,3^ | 190 (76.0) | 1.2 (0.8, 1.8) | 0.646 |  | 236 (78.4) | 0.8 (0.6, 1.0) | 0.158 | 1.6 (1.0, 2.6) | 0.162 | 0.03 | 0.26 |  |
| ***Stress*** |  |  |  |  |  |  |  |  |  |  |  |  |
| Perceived level of distress | 3.9 (2.7) | -0.5 (-0.9, -0.1)* | 0.158 |  | 4.0 (2.8) | 0.1 (-0.3, 0.5) | 0.880 | -0.6 (-1.2, -0.0)* | 0.158 | 0.00 | 0.21 |  |
| ***Health and Fitness Indicators*** |  |  |  |  |  |  |  |  |  |  |  |  |
| BMI (kg/m^2^) | 33.7 (9.1) | 0.4 (0.0, 0.7)* | 0.158 |  | 34.2 (8.8) | 0.3 (0.0, 0.6)* | 0.158 | 0.1 (-0.4, 0.5) | 0.898 | 0.02 | 0.01 |  |
| Waist circumference (cm) | 104.6 (18.6) | 0.2 (-1.1, 1.4) | 0.898 |  | 106.1 (17.9) | -0.1 (-1.1, 1.0) | 0.951 | 0.2 (-1.4, 1.8) | 0.898 | 0.01 | 0.01 |  |
| Mean arterial pressure | 94.2 (14.0) | -0.3 (-2.4, 1.8) | 0.898 |  | 95.0 (13.2) | -2.3 (-4.1, -0.6)* | 0.105 | 2.0(-0.7, 4.7) | 0.311 | 0.07 | 0.15 |  |
| 1 Square root transformation of the outcomes were used for analyses and thus providing valid p-values, however, results are presented in their original scale | | | | | | | | | | | | |
| 2 Results for baseline are presented as n (%) | | | | | | | | | | | | |
| 3 Results for change are presented as OR (95% CI)  4 Adjusted p-values accounting for multiple comparisons using the false discovery rate method  Unadjusted significance * p < 0.05, ** p < 0.01, *** p < 0.001.  PA = physical activity. MVPA = moderate-vigorous PA, CI = confidence interval, SD = standard deviation, ICC = intraclass correlation, ES = effect size | | | | | | | | | | | | |

| **Supplementary Table 2.** Results from intent to treat analyses for changes in child care centers’ workplace health and safety environmental supports from baseline to 18-month follow-up. | | | | | | | | | | | | | | | |  |
| --- | --- | --- | --- | --- | --- | --- | --- | --- | --- | --- | --- | --- | --- | --- | --- | --- |
|  | **Healthy Lifestyle**  **(n=28)** | | |  | | **Healthy Finance**  **(n=28)** | | | |  | |  | |  | |  |
|  | **Baseline** | **Change** | **Adj.** | |  | | **Baseline** | **Change** | **Adj.** | | **Diff in mean change** | | **Adj.** | |  | |
| **Outcome** | **mean (SD)** | **mean (95% Cl)** | **p-value^1^** | |  | | **mean (SD)** | **mean (95% Cl)** | **p-value^1^** | |  |  | **p-value^1^** | | **ES** | |
| ***Worksite Health and Safety*** | 41.8 (11.9) | -3.0 (-7.8, 1.7) | 0.436 | |  | | 44.0 (13.1) | -0.8 (-4.2, 2.5) | 0.733 | | -2.2 (-7.9, 3.5) | | 0.637 | | 0.18 | |
| Infrastructure score | 9.7 (4.2) | -0.9 (-2.8, 1.0) | 0.591 | |  | | 9.8 (3.7) | -1.8 (-3.1, -0.6)** | 0.048 | | 0.9 (-1.3, 3.1) | | 0.637 | | 0.23 | |
| Organization Policies & Procedures (OPP) | 11.5 (3.6) | 1.4 (0.1, 2.8)* | 0.114 | |  | | 12.3 (4.1) | 2.2 (0.5, 4.0)* | 0.064 | | -0.8 (-3.0, 1.3) | | 0.637 | | 0.21 | |
| OPP - Physical activity score | 1.6 (1.0) | 1.0 (0.5, 1.4)*** | 0.012 | |  | | 2.1 (1.3) | 0.9 (0.4, 1.4)*** | 0.012 | | 0.1 (-0.6, 0.7) | | 0.842 | | 0.06 | |
| Programs & Promotions (PP)^2^ | 7.4 (5.0) | -2.0 (-4.3, 0.2)* | 0.195 | |  | | 8.4 (5.8) | -0.2 (-1.6, 1.2) | 0.842 | | -1.8 (-4.4, 0.8) | | 0.382 | | 0.34 | |
| PP - Physical activity score | 0.5 (0.8) | 0.1 (-0.3, 0.5) | 0.733 | |  | | 0.5 (1.0) | 0.4 (0.1, 0.7)* | 0.064 | | -0.3 (-0.8, 0.2) | | 0.526 | | 0.33 | |
| Internal Physical Environment (IPE) | 13.2 (1.8) | -1.5 (-2.6, -0.4)** | 0.054 | |  | | 13.5 (2.2) | -1.2 (-2.2, -0.2)* | 0.069 | | -0.3 (-1.7, 1.2) | | 0.787 | | 0.00 | |
| IPE - Physical activity score | 0.7 (0.9) | 0.2 (-0.2, 0.6) | 0.591 | |  | | 0.9 (1.1) | 0.1 (-0.3, 0.5) | 0.707 | | 0.1 (-0.5, 0.6) | | 0.842 | | 0.00 | |

SD = standard deviation, CI = confidence interval, Adj. = adjusted

Unadjusted significance * p < 0.05, ** p < 0.01, *** p < 0.001.

1 Adjusted p-values accounting for multiple comparisons using the false discovery rate method

2 Square root transformation of the outcomes were used for analyses and thus providing valid p-values, however, results are presented in their original scale
